# Benign brainstem tumors in children: a retrospective series of 42 patients treated in Lyon

**DOI:** 10.1007/s00381-025-06908-z

**Published:** 2025-07-30

**Authors:** P. A. Beuriat, R. G. Boukaka, A. Szathmari, A. Vasiljevic, F. Di Rocco, C. Mottolese

**Affiliations:** 1 Department of Pediatric Neurosurgery, Hôpital Femme Mere Enfant, Hospices Civils de Lyon, Bron, France; 2 Université, Claude Bernard Lyon 1, Lyon, France; 3 Department of Neuropathology, Groupement Hospitalier Est, Hospices Civils de Lyon, Bron, France

**Keywords:** Brainstem tumors, Pediatric, Surgery, Chemotherapy, Radiotherapy, DIPG, Low-grade

## Abstract

**Introduction:**

Brainstem tumors represent 15% of brain tumors in children. Eighty percent of these tumors are diffuse pontine tumors with a globally poor prognosis even with the tailored chemotherapies and radiotherapy. Twenty percent of these tumors are benign and present a good prognosis with only a surgical treatment.

**Material:**

All patients admitted with the diagnosis of brainstem tumor in the Pediatric Neurosurgical Department between January 1997 and December 2021 have been considered for this study. We excluded patients with a diagnosis of DIPG or any other malignant histopathology.

**Results:**

Out of 112 patients with a brainstem tumor, 42 patients had a proved benign histological diagnosis and were included in this study. The average age, at diagnosis, was 8 years old, with a median age of 4.7 years. All patients were operated. Forty-eight surgical procedures were realized for the 42 patients: 8 biopsies and 40 direct approaches. Seventeen patients had a surgical treatment for hydrocephalus. Regarding surgical outcome, eight patients presented motor deficits, six improved, while in other two, the deficit was unchanged but not impeding the walk. The major clinical complication after surgery was cranial nerve palsy, present in 18 patients. Two patients deceased in the early postoperative period and two patients for a recurrence after 7 and 8 years after the diagnosis. Thirty-eight patients are alive.

**Conclusion:**

Benign brainstem tumors can be associated with a favorable evolution, a low growing rate, and, when symptomatic, curable with surgery. The surgical resection has to be safe and reasonable to avoid catastrophic complications. The results of surgery are particularly conditioned by the skills and experience of the surgeon.

## Introduction

Surgery of brainstem lesions represents an arduous challenge for neurosurgeons. “Brainstem” is a highly complex structure, housing numerous nuclei essential for somato-motor and somato-visceral functions, as well as major white matter tracts connecting the brain to the medulla, making any surgical intervention inherently high-risk [1, 2].

Brainstem tumors account for 15% of children brain tumors, and 80% of these tumors are diffuse intrinsic pontine gliomas (DIPG) that have a dismal prognosis. This also means that 20% have a more favorable prognosis [3–5].

Pediatric low-grade brainstem tumors are more likely to be well-circumscribed, associated with better prognosis. Brainstem low-grade gliomas can be classified by location, as tectal midbrain, tegmental midbrain, focal pontine, pontomedullary, medullary, and cervico-medullary [2, 6, 7]. Several articles have been published on brainstem anatomy, reporting safe entry zones to facilitate their removal. Most of these studies are related to cavernoma surgery, and few are related to brainstem tumor surgery in children [8–11].

We have already reported our experience with brainstem tumors [3], but we want to focus particularly on the treatment of benign lesions treated in the Pediatric Neurosurgical Department of Lyon (France).

## Materiel and methods

All patients admitted with a diagnosis of brainstem tumor in the Pediatric Neurosurgical Department at the “Hôpital Femme Mère Enfant” (Hospices Civils of Lyon, Lyon, France) between January 1997 and December 2021 were considered for this study. We excluded patients with a diagnosis of DIPG or any other malignant histopathology. We also excluded patients without a clinical history, radiological images, and without a documented postoperative follow-up.

The extent of surgical removal was judged by the neurosurgeon who performed the surgery and by the postoperative MRI realized in the first 48 h after the procedure [12]. The extend of resection was definite as follows: complete resection, no residual tumor visible on the postoperative MRI; near total resection, when residual tumor was visible < 10% of the initial volume; sub-total, when less than 75% of the initial volume was removed; partial, when < 50% of the tumor was removed; and biopsy, when less than 25% of the initial volume removed [12].

The study protocol was approved by the local ethics committee. Of 112 patients treated for a brainstem tumor, only 42 patients had a proved benign histological diagnosis and were included in this study.

## Results

**Fig. 1:**
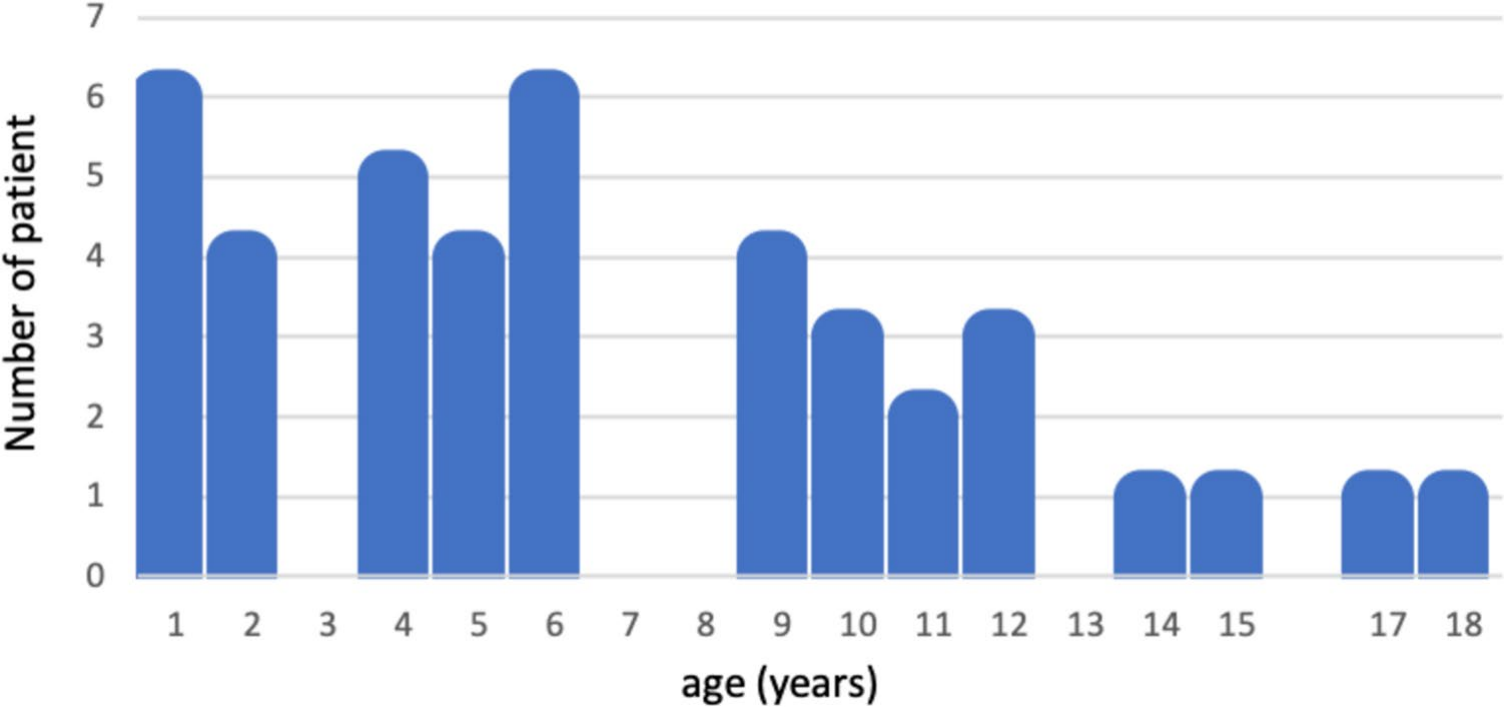
Bar graph depicting the age distribution of the population

Demographic of the study population is detailed in Table 1. Age distribution is detailed in Fig. 1. The follow-up varied from 25 to 3 years, with a median 11.3 years.

**Table 1:** Demographic of the study population

Age at diagnosis	
Mean (y)	8
Median (y)	4.7
Range	9 m–18 y
Sex	*N* (%)
Male	19 (45.2%)
Female	23 (54.8%)
Histology	
Ganglioglioma	11 (26.2%)
Gangliocytoma	2 (4.8%)
Pilocytic astrocytoma	17 (40.5)
Low-grade glioma	10 (23.8%)
Glioneuronal tumor	2 (4.8%)
Surgery	
Hydrocephalus	17 (40.5%)
Tumor surgery	48
Adjuvant therapy	
Chemotherapy	17
Radiotherapy	7
Overall survival	38 (90.4%)

The clinical symptomatology was characterized by signs of intracranial hypertension in 10 patients (23.8%) and cranial nerve palsy in 11 patients (26.1%), balance disorders in 5 patients (12.12%). Motor and sensitive deficits were also present in eight patients (19%), concentration troubles associated with weight loss in four patients (9.5%), anorexia in three patients (7.1%), and in one patient, the discovery was done after a cranial trauma.

Regarding the cranial nerve’s involvement, the distribution of the deficit was as follows: 3rd nerve (*n* = 1), 5th nerve (*n* = 1), 6th nerve (*n* = 2), 7th nerve (*n* = 2), 8th nerve (*n* = 1), 9th and 10th nerve (*n* = 2), and 11th nerve (*n* = 2).

**Fig. 2:**
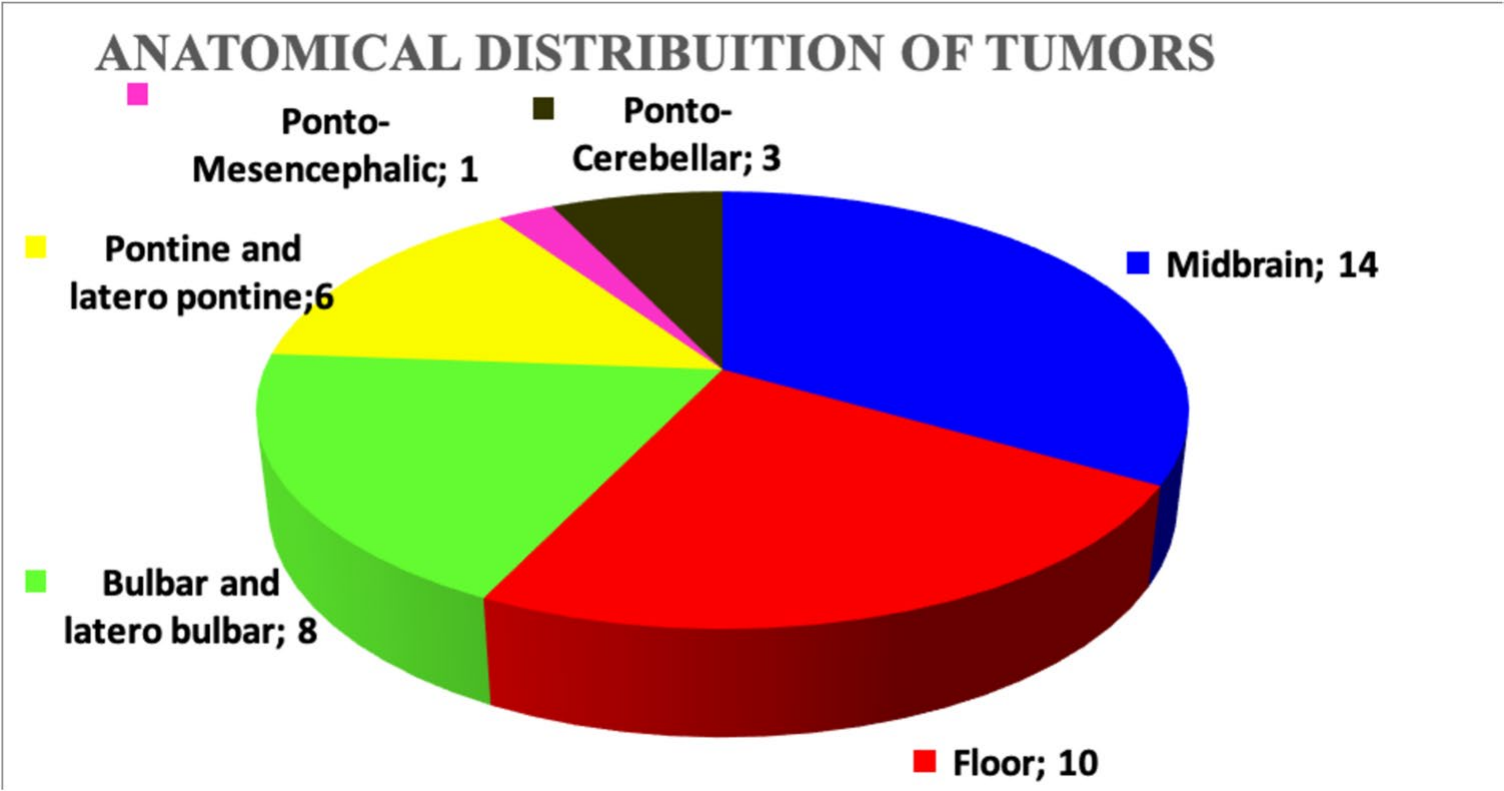
Pie chart depicting the anatomical localization of the tumors

All patients underwent a preoperative cranio-spinal MRI both without and with gadolinium. The localization of tumors is described in Fig. 2.

Five patients presented an extension of the tumor in the cerebellar hemisphere and four patients in the cerebello-pontine angle. An extension inside the fourth ventricle was observed in 28 patients.

All 42 patients were operated. A total of 48 surgical procedures were performed consisting of 8 biopsies and 40 direct tumor approaches. One patient treated with an ETV without biopsy and followed clinically and radiologically yearly was excluded from this series.

### Surgical outcomes and procedures (Table 2)

**Table 2:** Type of surgery, surgery outcome, and complications

	***N*** ** (%)**
Surgery	
Hydrocephalus	17 (40.5%)
Shunt VP	11 (64.7%)
ETV	6 (35.3%)
Type of surgery	
Tumor surgery	48
Biopsy	8 (16.7%)
Open	2 (25%)
Stereotactic	4 (50%)
Neuronav ass	2 (25%)
Direct approach	36 (75%)
Total	14 (38.9%)
Total after second look	2 (5.5%)
Sub-total	13 (36.1%)
Partial	7 (19.4%)
Re operation	4 (8.3%)
Perioperative complications	
Cerebellar contusion	1 (2.4%)
Postoperative complications	
Cranial nerve palsy	18 (42%)
Motor deficit	8 (19%)
Balanced disorder	6 (14%)
Beauty sleeping sd	1 (2.4%)

Total tumor resection was achieved in 14 patients (33.3%), while 13 patients (30.9%) underwent subtotal resection, and 7 patients (16.6%) had partial resection. In two patients (0.05%), the resection was total after a second-look surgery. Four patients were operated again after a partial removal and after a complementary chemotherapy.

Biopsies were performed using different techniques:

- Open surgical biopsy in two patients.

- Neuronavigation-assisted biopsy in two cases prompted by MRI findings indicating rapid tumor progression after an initial subtotal surgery.

- Stereotactic biopsy using the ZD system in four cases.

Concerning patients treated for a hydrocephalus, a VP shunt was realized in 11 patients and an ETV in 6 patients. In eight patients, the shunt was realized before the biopsy or before the surgical approach of the lesion, while in three other cases, it was realized after the surgical resection for a postoperative acute hydrocephalus. We never used external temporary ventricular drainage.

### Perioperative complications (Table 2)

One patient presented a perioperative cerebellar diffuse edema after the closure of the dura mater that led to a partial cerebellar resection of a contusion area on the right cerebellar hemisphere. A dural graft and the removal of the bone flap were realized, and the patient was placed in a pharmacologically induced coma for 48 h to prevent postoperative intracranial hypertension. The patient progressively woke up with a cerebellar ataxia that improved progressively in the following 6 months when she was operated again for a cranioplasty of the posterior fossa with her autologous bone flap.

A patient presented with cerebral ischemia after a perioperative air embolism; however, no clinical sequelae were observed.

### Postoperative clinical complications (Table 2)

The most frequent postoperative clinical complication was represented by cranial nerve palsy affecting 18 patients (42%). Multiple nerve deficits could be present in the same patient. Distribution included:

- Facial nerve (VII) palsy in four patients (22%).

- Abducens palsy (VI) in six patients (33.3%).

- Oculomotor nerve (III) in three patients (16.6%).

**-** Mixed nerves (IX, X, XI) involvement in eight patients (44.4%); two patients required temporary tracheotomy.

Eight patients (19%) presented a motor deficit: six improved over time while two retained persistent but non-disabling deficits that did not interfere with deambulation. One patient (2.4%) had a prolonged beauty sleeping syndrome, and six (14%) had balance disorders.

### Histopathology

The histological diagnosis revealed a grade I ganglioglioma in 11 patients, a gangliocytoma in 2 patients, a pilocytic astrocytoma in 17 patients, an astrocytic low-grade glioma in 10 cases, and a glioneuronal tumor in 2 cases. The localization for each is indicated in Table 3.

**Table 3:** Details of the histology for each tumor’s anatomical localization

Localization of the tumor	*N* (%)	Histology: *n*
Tectal plate	14 (33.3%)	Pilocytic astrocytoma: 9 Ganglioglioma: 2 Low-grade astrocytoma: 3
Bulbo-medullary joint	16 (38%)	Pilocytic astrocytoma: 6 Gangliocytoma: 2 Ganglioglioma: 4 Low-grade astrocytoma: 5
Peduncular	2 (4.7%)	Low-grade astrocytoma: 1 Pilocytic astrocytoma: 1
Latero-pontine	6 (14.2%)	Low-grade astrocytoma: 3 Ganglioglioma: 3
Ponto mesencephalic	1 (2.4%)	Pilocytic astrocytoma: 1
Ponto cerebellar	3 (7%)	Low-grade astrocytoma: 1 Ganglioglioma: 2

A biomolecular and genetic study was realized in 32 patients. In six patients, with a ganglioglioma, a positivity for the BRAF V600E mutation was found. In four patients, with a pilocytic astrocytoma, the transcript KIAA1549-BRAF fusion was present.

The GFAP was positive in 66% of the tumors, the Olig 2 in 37%, and the ATRX in 3.7%. The P53 was low in 18.5% and between 3 and 15%; the Ki67 was between 2 and 10% in 44.4%. No tumor shoved mutation of histone H3K27m.

### Progression (Table 4)

**Table 4:** Management of tumor progression and relapse

	*N* (%)
Total number of patient	7 (16.7%)
Number of re operation	4 (64.7%)
Localization	
Latero-bulbar	2 (50%)
Bulbar	1 (25%)
Tectal plate	1 (25%)
Histology	
Pilocytic astrocytoma	3 (75%)
Ganglioglioma	1 (25%)
Type of surgery	
Total	3 (75%)
Open biopsy	1 (25%)
Adjuvant treatment	
Chemotherapy	4 (100%)
Number of patients followed (without re operation)	3
Localization	
Pontine	1 (33.3%)
Bulbar	1 (33.3%)
Ponto-cerebellar	1 (33.3%)
Adjuvant treatment	
None	4 (100%)

A tumor progression occurred in seven patients.

Four patients were reoperated: the location was latero-bulbar in two cases, bulbar in one case, and at the level of the tectal plate in one case. The first surgery was judged total in three cases and was subtotal in one case. The histological diagnosis was a pilocytic astrocytoma in three cases and a ganglioglioma in one case.

The second surgery was again complete in three cases and a simple large biopsy in one case. In ganglioglioma recurrence, the genetic studies shoved the mutation BRAF6V600E, and the KIAA1549-BRAF fusion transcript was expressed in the pilocytic astrocytoma.

In all surgical cases, a chemotherapy was realized.

### Mortality

Two postoperative deaths were observed after 5 and 8 months after surgery for respiratory complications.

Two patients for a tumoral progression were treated with chemotherapy and radiotherapy. A new biopsy performed 7 and 8 years after the initial diagnosis revealed a malignant transformation. The genetic studies did not show a mutation of the histone H3.3 (H3K227M).

Both patients succumbed to disease progression within the same year.

**Fig. 3:**
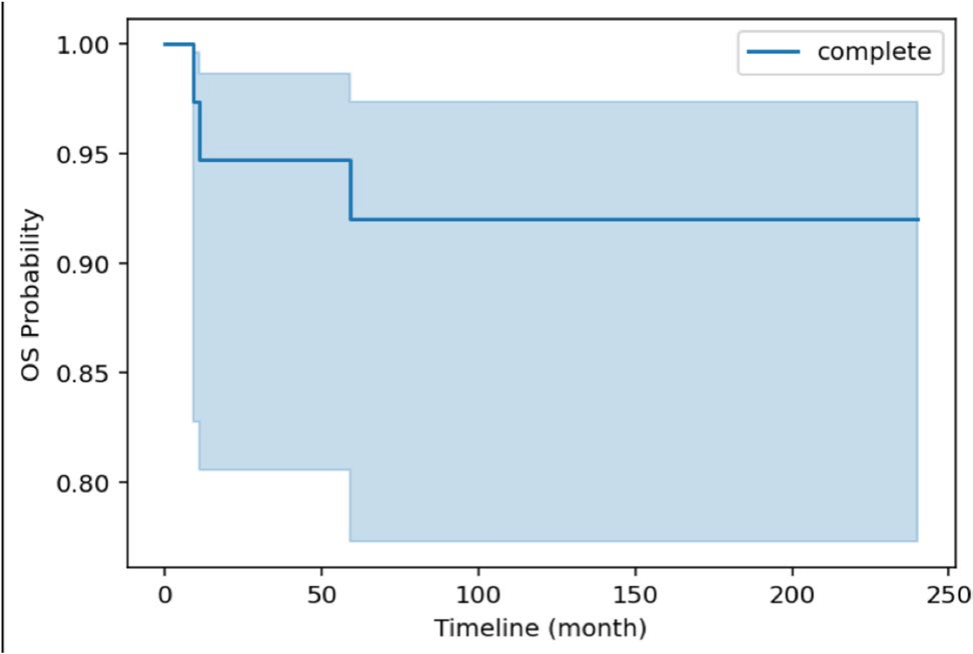
Overall survival curve of the Lyon Series

Consequently, the survival rate was of 90.4% and the rate of mortality was 9.6% (Fig. 3) (Table 3).

### Tectal plate tumors results

**Fig. 4:**
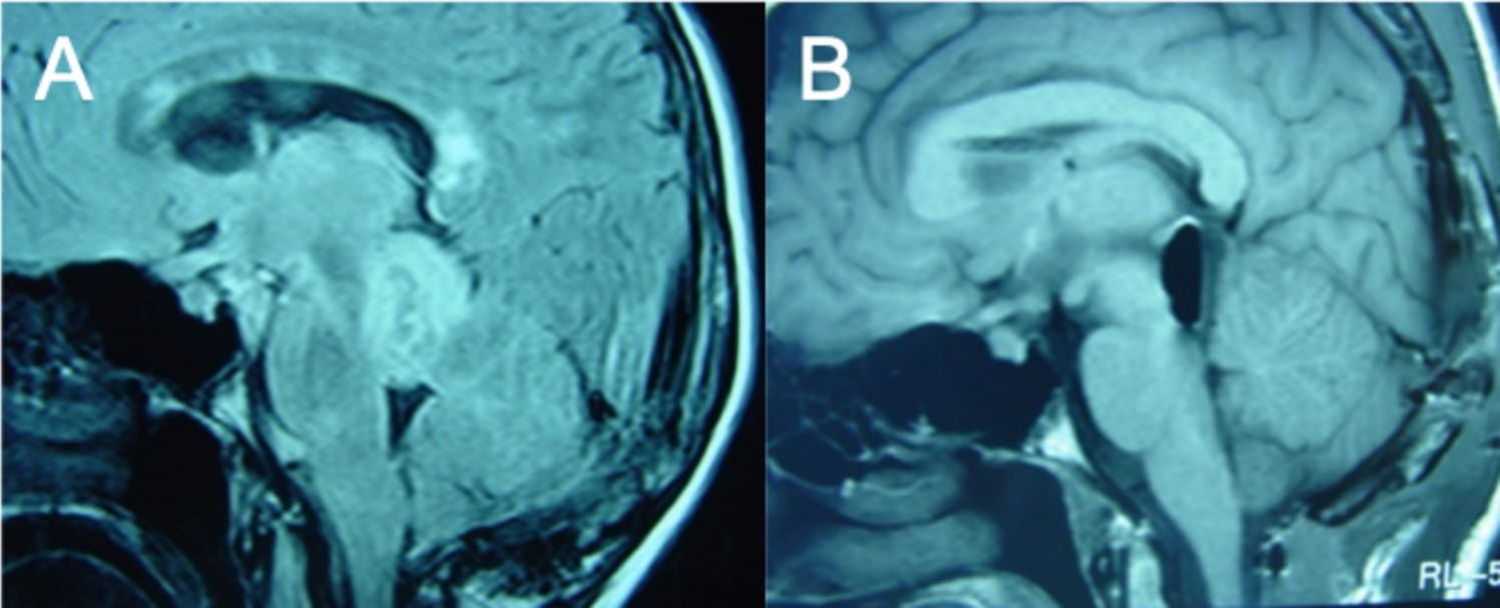
Preoperative (**A**) and postoperative (**B**) T1-weighted sagittal MRI scan of a tectal plate tumor

Fourteen patients with tectal tumors were WHO grade I tumors: nine (64.2%) pilocytic astrocytomas, two (14.2%) gangliogliomas, and three low-grade astrocytomas (21.4%). All tumors were exophytic and consequently all of them benefited from an aggressive resection (Fig. 4).

The resection of pilocytic astrocytoma, with well-delimited tumoral plane, makes surgery easier to attain a complete removal instead of the different behavior of ganglioglioma or low-grade astrocytoma that do not present a clear cleavage plane, with an increased risk of leaving tumors in place and an increased risk of sequels to obtain a complete resection [13].

## Discussion

Brainstem low-grade gliomas are rare childhood brain tumors and, despite their benign histopathology, have the potential to cause significant neurologic morbidity [12, 14, 15].

Whenever feasible, surgical gross total resection or safe maximal resection has been proposed as the treatment [16]. Several studies reported sustained disease control after complete tumor resection; as our results indicate, no recurrences occurred among patients with a total resection [16].

Favorable results have been reported for low-grade brainstem gliomas when an extended resection is realized versus a simple biopsy [17].

Low-grade brainstem tumors are slow-growing tumors with in many times indolent course and a favorable prognosis. Consequently, the policy of “watching and see” can be justified because the potential growth is estimated to be only 4 mm in tumor diameter for year [12].

A retrospective review of 96 patients treated at Hospital for Sick Children in Toronto reported a favorable outcome after upfront observation as a first-line management in some patients with brainstem low-grade gliomas and significant residual tumor after resection [4, 18].

A very good progression-free survival of 70% was similarly noted for cervico-medullary low-grade gliomas following surgical resection alone, by Robertson et al. [4]. Lundar et al. demonstrated a favorable 47% progression-free survival in 15 patients with low-grade midbrain tumors that were surgically resected upfront [13].

No further adjuvant therapy or repeat resection was required in these patients, the majority of whom had not undergone a total resection [4, 19].

Thus, a very close surveillance can be a safe approach for many brainstem low-grade gliomas with residual tumor after the diagnosis. However, a significant fraction of these tumors can progress and require additional treatment, for which both chemotherapy and radiotherapy have been effective therapies, also if sometimes a safe surgical resection can be evaluated and realized [9, 20].

### Surgery

Surgery has been indicated as the treatment of choice whenever possible because the potential risk of surgical sequels must be weighed against the potential risk of natural evolution, particularly with regard to the anatomical location in the brainstem.

When surgery is indicated, it demands experience and a great ability to avoid mortality and to reduce the risk of morbidity and postoperative complications.

The brainstem has been divided into different areas that can be approached by several entry zone points as reported by different authors in literature [2, 8–10].

While knowledge of the precise location of the safety entry zone is critical at all brainstem levels, the practical surgical objective is often to select the route with the smallest distance from the normal structures and where tumors present the exophytic component. The exophytic portion of the brainstem tumor can be generally removed without violating functional structures [2, 21].

It is important to avoid the nuclei of the cranial nerves at different levels as the long motor and sensitive fibers to prevent motor and sensitive deficits, or whenever already present, to reduce the risk of their aggravation.

For surgery of focal tectal plate tumors, the resection of one colliculus may be performed on the condition that contralateral structures are preserved. In larger lesions, an extended resection is possible without going beyond the plane of the aqueduct [19, 21]. Surgery of tectal plate tumors was reported in the 1990 s by Professor Lapras [19, 22].

Sometimes, after an extended resection in the tectal plate, a phenomenon described as “beauty sleeping syndrome” may occur. This syndrome typically resolves progressively as reported already in Lyon [19, 21, 23].

The surgical resection is facilitated by:

- Availability of high-performance ultrasonic aspirator with handpiece of different lengths according to the depth of the surgical field.
- An effective bipolar forceps to obtain a rapid control of hemorrhage.
- Intraoperative neuronavigation for monitoring the surgical resection and to avoid functional structures.

The use of per-operative fluorescein can be useful to increase the tumoral volume resected.

The choice of the approach depends on the precise location of the tumors and its development, whether intra-axial or exophytic.

### Midbrain lesions (16 patients)

The approaches can be anterior or posterior or postero-lateral in function of the origin of the tumor and its axis of development.

A pterional transylvian approach was used in two cases of anterior or antero-lateral mesencephalic peduncular location.

For low-lying tumors, the orbito-zygomatic approach enhanced the surgical corridor even if the exposure remains limited due to the presence of the vascular structures and the third cranial nerve. The approach to the tumor has to be done where the tumor is more superficial, paying attention to the instruments when they are introduced and when they leave the operative field to avoid contusion of the healthy tissue and avoid lesion of the third nerve and arterial feeders. Particular attention needs the use of the cavitron that has to start its suction when the tip is inside the tumor to avoid damage to the superficial tissue.

Posterior midbrain tumors (14 cases) were approached using the suboccipital transtentorial route, which offers a large vision of the tectal plate, the pineal region, and the venous arch formed by both the basilar veins and the Galen vein [21].

In our experience, as already reported by C. Lapras, it is important to know the limits of the resection when the tectal plate is fully involved. The resection should not surpass the plane of the aqueduct superiorly, or inferiorly the plane passing through the encroachment of fourth nerves [19, 21].

We previously reported a cohort of 27 pediatric patients treated successfully with good results using these guidelines [21].

Nowadays, also for tectal plate tumors, the genetic studies help to individualize favorable prognostic factors and tailored chemotherapy to control the disease. In two patients with a recurrence, a second surgery shoved a more aggressive tumor treated with a protocol of chemotherapy.

It is important to perform molecular studies to enhance the diagnostic precision of biopsies or of surgery supporting the histological diagnosis. Also, the MRI, during the follow-up, can help to discover a malignant progression, with a modification of a contrast enhancement or a growth progression that could need a new biopsy. The surgical resection of tumors in this region is, in our experience, simpler for pilocytic astrocytomas than for gangliogliomas because, in the former, there is a cleavage plane which favors the surgeon and the excision. In case of gangliogliomas, the absence of a cleavage plane favors the risk of deficits in case of pushed resection.

### Pontine lesions (10 patients)

**Fig. 5:**
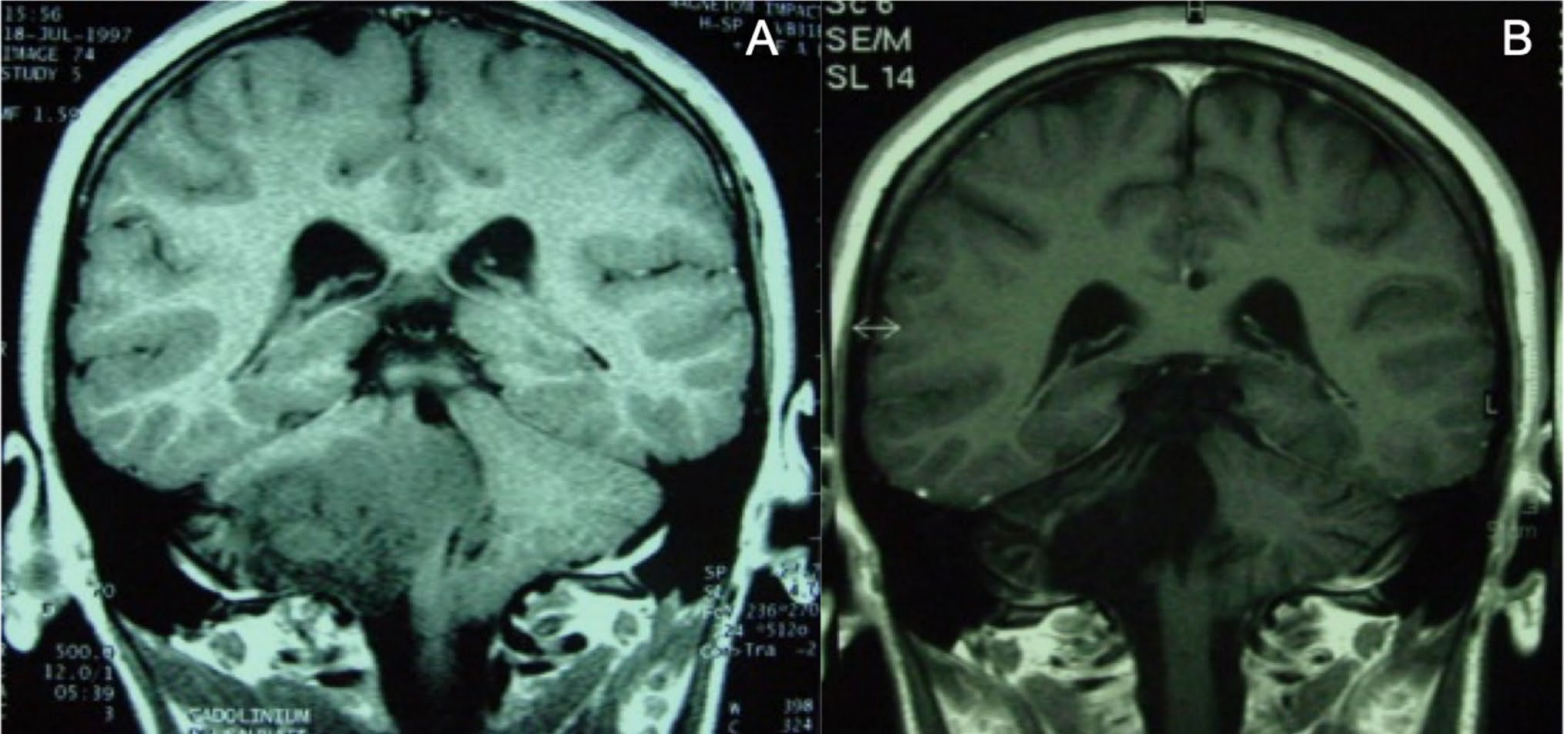
Preoperative (**A**) and postoperative (**B**) T1-weighted sagittal MRI scan of a ponto-cerebellar tumor

For pontine lesions with extension in the cerebello-pontine angle, we used a lateral approach through the cerebello-pontine angle (Fig. 5). Posteriorly located or focal lesions were treated via a postero-medial trans ventricular approach.

**Fig. 6:**
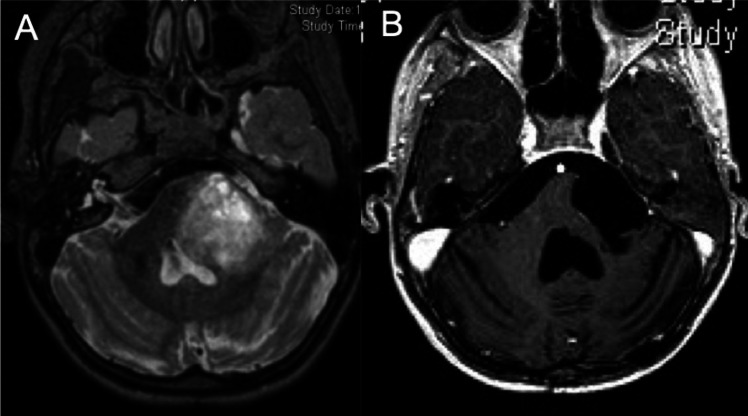
Preoperative (**A**) T2-weighted and postoperative (**B**) T1-weighted sagittal MRI scan of a latero-pontine angle tumor

For lateral pontine location (Fig. 6), the subtemporal approach can be useful because it allows a large vision and a good control of the tumoral resection.

With the sub-temporal approach, problems can be represented by the compression to retract the temporal lobe, with a possible venous ischemia, and by a fourth cranial nerve injury related to the opening of the tentorium posterior to the fourth nerve [24, 25].

### Bulbo-medullary lesions (16 patients)

For tumors involving the bulbo-medullary junction, the median approach trans the posterior sulcus was the approach more frequently employed, particularly in cases with an exophytic extension in the fourth ventricle.

**Fig. 7:**
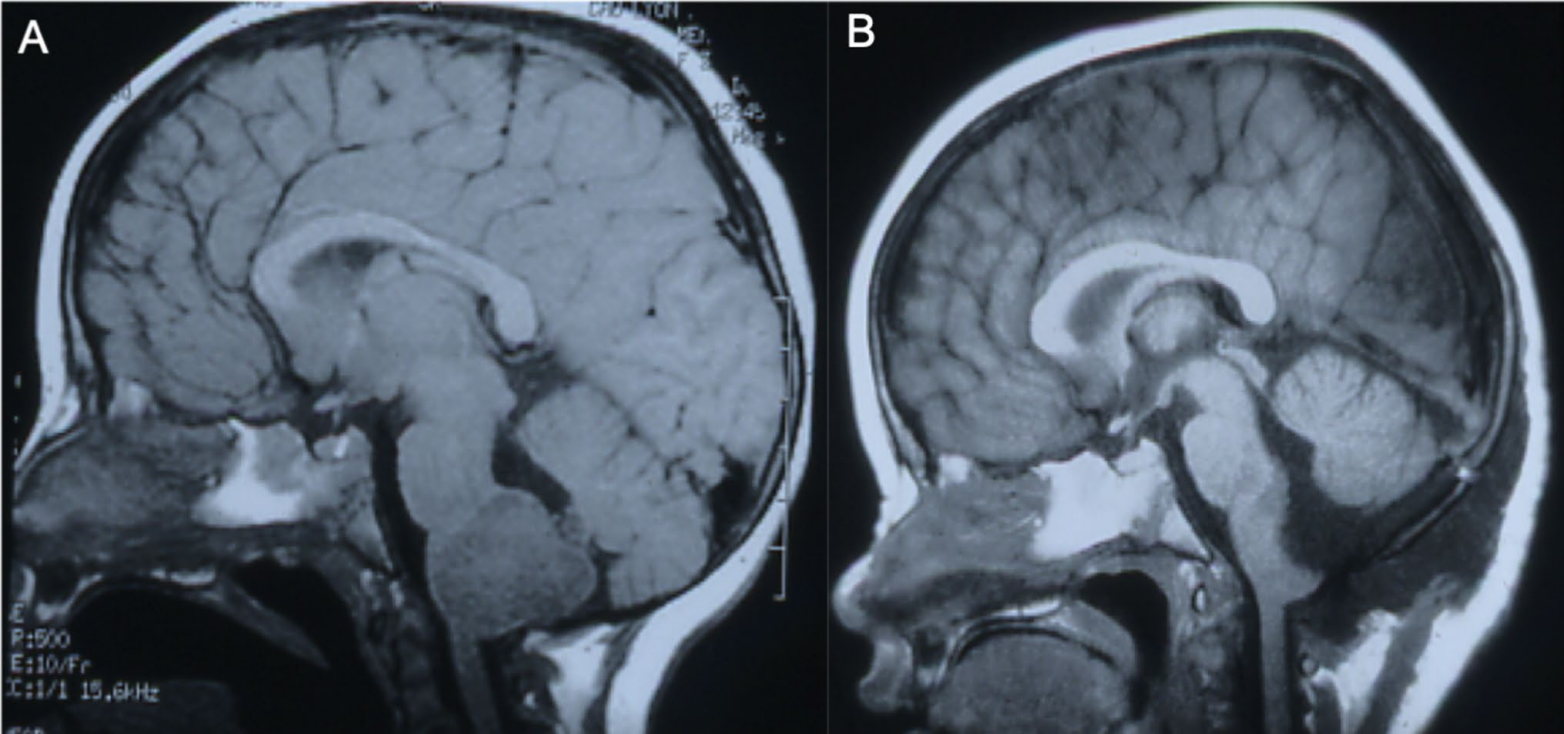
Preoperative (**A**) and postoperative (**B**) T1-weighted sagittal MRI scan of a bulbo-medullary junction tumor

The extension of the resection was tailored in function of the vascularity of the tumor, the size of the exophytic portion, and the presence or absence of a well-defined plane around the tumors (Fig. 7).

In cases of brainstem tumors with extension in the cerebellar hemisphere, the resection could be extended until the brainstem was joined. Inside the brainstem, the resection has to proceed with caution, respecting the idea of preserving functional structures (Fig. 4).

We have to keep in mind that after the resection, frequently, benign brainstem tumors can remain silent for a long time and, sometimes, a simple “wait and see “ strategy can be appropriate because low-grade tumors are frequently indolent and have a low growth rate [16].

In our series, a total removal was achieved in 16 patients (40%), a subtotal resection in 12 patients (27.6%), and a partial resection in 7 patients (23.4%). These results are consistent with reported standards of literature [16].

**Fig. 8:**
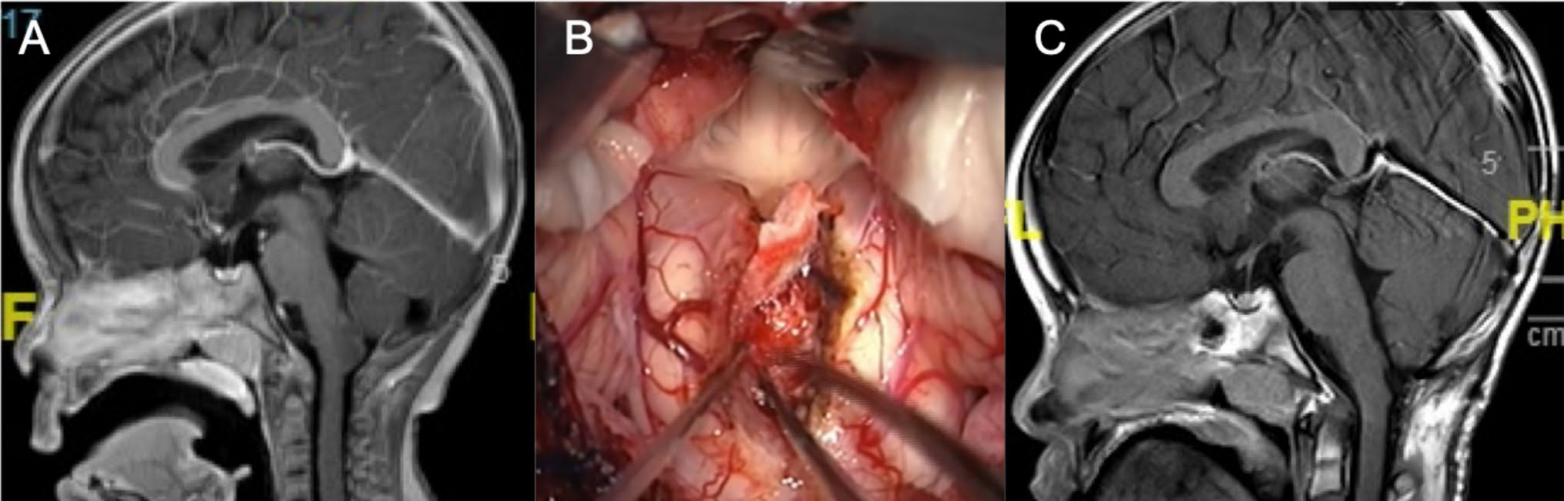
Preoperative (**A**) and postoperative (**B**) T1-weighted sagittal MRI scan of a bulbar tumor with per-operative microscopic view (**C**)

The neurophysiological monitoring, neuronavigation, and tractography are important tools; however, to prevent deficits, we believe that the most important factor is to avoid intempestive coagulation of arterial or venous structures, which can result in devastating complications and severe sequelae (Fig. 8).

Despite the multiple entry zone described in literature, the key surgical principle, to remove tumors in these difficult regions, is to remain inside the tumors and to respect the vascular supplies. Given the small superficial couloirs, it is always challenging to recognize nuclei or nervous functional fibers to reduce the incidence of deficits. Continuous electrophysiological monitoring is therefore essential to reduce postoperative morbidity.

### Postoperative morbidity and mortality

The rate of sequels, in our series, was of 38% primary involving cranial nerve palsy as observed in 16 patients in the early postoperative period.

Mixed nerves (IX, X, XI) were the most affected nerves, resulting in temporary tracheotomy in two patients that was possible to remove within 6 months.

A palsy of the facial nerve (VII) occurred in four patients, regressed in two patients, and was permanent in the other two.

Abducens nerve palsy (VI) was diagnosed in six patients and was permanent in two who needed a surgical correction to improve the strabismus.

At the follow-up, the cranial nerve deficits persisted in only eight patients (20%).

Motor and cerebellar deficits were noted in 15% of patients but did not impair the walk and also the activity in daily living.

Early postoperative mortality was, after 6 and 8 months from surgery, of 4.7% and definitively of 9.5% after a follow-up of 8 years because two patients deceased for an evolving disease at the level of the ponto-bulbar junction despite adjuvant chemotherapy.

In Cavalheiro’s series, the rate of sequels was of 21% while the rate of mortality was of only 1.9% but related only to an early postoperative period [2].

### Management of hydrocephalus

Concerning the treatment of hydrocephalus, in our long-lasting review, 11 patients needed a VP shunt and only 6 patients definitively were treated with an ETV.

The ETV provided a route for biopsy of some extrinsic tumors with an anterior development in the interpeduncular cistern [26], but this option was used only in one of our cases and for tumors bulging in the third ventricle, two cases in our series.

For tectal plate tumors, on the contrary of pineal tumors, we do not think that the endoscopic biopsy through the third ventricle can be recommended because tectal tumors do not develop inside of the third ventricle but push the normal posterior wall inside the ventricle and, consequently, the biopsy forceps have to cross healthy structures with the risk to increase deficits and the possibility to obtain normal tissue instead of pathological material.

### Biopsy techniques

For brainstem biopsy, we prefer actually to use the Medtronic neuronavigation-guided biopsy system that, in our experience, was sure, effective also in very young patients. It allows to obtain multidirectional sampling by rotating the needle by 90° for biopsy in the orthogonal plane of the space [3].

Brainstem tumors biopsy remains a challenging procedure [27] also if associated with a low risk as reported in the literature [17, 28]. The rate of morbidity was evaluated at < 2% and the efficacy at a rate of 94% of cases [26].

Recently, robot-assisted stereotactic biopsy has been used with favorable results and a low rate of complications [29].

### Chemotherapy and radiotherapy

Chemotherapy is effective as adjuvant therapy against relapse/progression of brainstem low-grade gliomas, and as salvage therapy for recurrent or progressive disease [12, 16]. In such situations, chemotherapy may eliminate the risk of surgical resection or repeat surgical interventions and can defer radiotherapy in young children until a more appropriate age when potential adverse effects are better tolerated.

Chemotherapy seems particularly advantageous for treatment of tumors in young children with challenging surgical tumors, or in those presenting neurologic deficits that may worsen with further tumor growth [30].

Nevertheless, given the generally favorable prognosis of these tumors, sometimes even after minimal surgical resection and without adjuvant therapy, the potential long-term morbidity of chemotherapy must be carefully considered, establishing the treatment plan. Molecular characterization of these tumors is increasingly valuable for selecting targeted therapies with the goal of minimizing adverse effects of complementary therapy.

In our series, 12 patients were treated with chemotherapy in eight cases as adjuvant treatment and in four cases after MRI confirmed a disease progression.

The association with vincristine and carboplatin was used in eight patients, BCNU with cisplatinum in a patient, oncovin and carboplatin in a case, and a protocol with only vinblastine was realized in two cases. All treatments were decided by our oncological team, in neuro-oncological meetings, in collaboration with the protocols of the French Society of Pediatric Oncology.

In one case, the treatment was stopped for hematological toxicity.

Seven patients were treated after chemotherapy with radiotherapy, and in one patient, the irradiation was realized after a new look surgery followed by additional chemotherapy.

The profiling and genomic studies of gliomas, as in other tumors, give the possibility to improve the prognostic factors that condition the good evolution of patients. The choice of drugs for targeted treatment can improve the evolution and the survival of patients [30].

In benign gliomas, genetic modifications are present in different percentages. In our series, the mutations V600-BRAF and the KIAA1549-BRAF fusion were detected in gangliogliomas and in pilocytic astrocytomas. The weight of genetic studies’ progress, in our series, is difficult to establish because these studies became routine seulement after 2015, and our series is over 20 years old.

The role of radiotherapy is well established in the literature in managing low-grade gliomas, although concerns regarding sequels in pediatric patients remain, also if attenuated by the fact that the irradiation is directed mainly in the posterior fossa with mild adverse effects [31] [32].

In the last time, proton therapy has been proposed because it is associated with further reduction of toxicity, making it an attractive option [33, 34].

For Indelicato, the rate of toxicity for brainstem irradiation is higher than photon therapy, varying between 0 and 10% [35].

The toxicity for treatment of brainstem tumors either with chemotherapy and either with radiotherapy, in the context of slow-growing or indolent tumors, has to push to postpone the decision for complementary treatments.

### Survival and quality of life

The rate of survival in our series of benign brainstem tumors was 90% with a rate of mortality of 10% (four cases, two cases after surgery and two patients after 6 and 8 years for the evolution of the disease).

Seventy-two percent of patients achieved a survival superior to 10 years, and 94% a survival superior to 5 years.

The outcome is consistent with literature reports for benign brainstem tumors, with a survival of 90% at 5 years and 75% at 10 years, with better prognosis for WHO grade I tumors compared to grade II [15].

### Educational and functional outcomes

The quality of life was primarily evaluated through the Ped QL, the EORTC, for some patients in age superior to 18 years in the follow-up, and with the education attainment and the professional integration. The Ped QL evaluation defined two groups of patients with a score between 60 and 81 and a group between 81 and 95 with a globally good quality of life.

The favorable results of the evaluation of quality of life explain, in our opinion, the good scholarly results.

Twenty-five patients were in the school program: four with a high school diploma, four patients in elementary school, six patients attended middle school, and three patients’ technician senior diploma.

Four patients followed a normal school program but with an academic school support.

Four patients developed a clinical brainstem-related neurobehavioral symptoms characterized by explosive behavior with irritability, memory impairment, attention deficits, and pathological laughter or crying.

The symptoms improved during the treatment [16] but did not resolve completely [36].

Recently, Jia documented impairment of the behavioral emotional cognition in children in a larger series of patients studied with the children behavioral checklist (CBCL) [36].

These observations emphasize the importance of neuropsychological investigations to better understand the mechanism responsible, at different anatomical levels of the brainstem, for the clinical neuropsychological problems.

In some patients, the cerebellar per-operative stimulation to establish a cartography of the cerebellum revealed interesting phenomena such as the overlapping of the mouths and the hand, the mouth and the tongue, the segmentary representation of the arm on the foot, or the possibility to release movements of the jaw and of the lips [37, 38]. How these data can explain neuropsychological problems is not yet clear but sufficiently intricating for us to continue our explorations.

The brainstem is regulated by the mediators such as the norepinephrine, dopamine, and serotonin that coordinate the pathways of behavior, cognition, and emotion projecting in the cerebellar area, the cortical cerebral region through the fronto-pontine loop, the cortico-ponto-cerebellar circuit, and the cerebello-thalamic-cortical pathways. We must not forget the amigdalo-hypothalamus-periaqueductal-ventromedial hypothalamus.

Our cohort was restricted to only patients with WHO classification of grade I and II tumors; it is certainly a limitation but strengthens the fact that the evolution of benign brainstem tumors can be favorable and also associated with a long survival, as confirmed by our series that concerned in 67% of cases the diagnosis of pilocytic astrocytoma and ganglioglioma of grade I.

Despite the good evolution, it is necessary also to have a very long follow-up because two cases of our series experienced a malignant transformation 8 and 9 years after the diagnosis but were unresponsive to a chemotherapy.

A malignant transformation after a long-term follow-up was reported by Maertens in tectal plate tumors in adult patients [39] while in the series of Upadhyaya, 8 patients (32%) of the 25 reported presented a progressive disease at 5 months of the diagnosis, with a range of 3 months to 16 years.

## Conclusion

Benign brainstem tumors constitute a distinct group of tumors associated with a favorable evolution, a low growing rate, and, when symptomatic, a curable with surgery, on the contrary of what happens with high-grade lesions, which still actually have an unfavorable prognosis.

Surgery in exophytic form or in focal localized tumors can attain a surgical total resection in 68% of cases with a potential cure and an acceptable rate of sequels evaluated, in the more recent series, at 20% of cases [2, 12, 16].

The surgical resection has to be safe and reasonable to avoid catastrophic evolution, but it is particularly related to the surgical skills of surgeons who have to use all the technical tools that can help for the surgical resection. The discussion in oncological pediatric concertation staff is necessary, but it is always difficult to choose between the risks related to surgery and the risks related to the natural history of tumors and the risks of complementary treatments and their collateral effects, either for chemotherapy or radiotherapy [33, 34, 40].

In some cases, the removal of tumors can be done with the dissection in a surgical plan as in cases of tectal plate tumors or remaining in the tumoral bed with the help of the Cusa cavitron that permits the progressive removal through a very small entrance door.

It is also impossible to predict the consequences of coagulation of intratumoral vessels responsible for ischemic lesions.

Until the discovery of more effective targeted chemotherapy of benign brainstem tumors, a reasonable surgical resection has to be the treatment of election for tumors that present exophytic development or evolutive growth.

Surgery can be also useful in cases of recurrences, as in four cases of our series, followed in two cases by a treatment with chemotherapy.

Consequently, for us, the decisional tree for benign brainstem tumors could be the following:

- For asymptomatic tumors a simple follow-up with yearly MRI.

- For tumors with hydrocephalus, ETV, or a shunt ± biopsy.

- For focal tumors resection surgery with a reasonable safe resection.

- For tumors with a subtotal resection “wait and see” or second look surgery with chemotherapy followed, in patients older than 7 years, by radiotherapy

## Data Availability

No datasets were generated or analysed during the current study.
